# Oral self-care behavior and its influencing factors in a sample of school children from Central Iran

**DOI:** 10.1186/s13690-021-00695-0

**Published:** 2021-10-12

**Authors:** Iraj Zareban, Mahmood Karimy, Marzieh Araban, Daniel Delaney

**Affiliations:** 1 Health Promotion Research Center, Zahedan of University Medical Sciences, Zahedan, Iran; 2 Social Determinants of Health Research Center, Saveh University of medical sciences, Saveh, Iran; 3 Department of Health Education and Promotion, Social Determinants of Health Research Center, Public Health School, Ahvaz Jundishapur University of Medical Sciences, Ahvaz, Iran; 4 Clinical Psychology Department, PhD. Student University of Rhode Island, New England, USA

**Keywords:** Oral health, Self-care behavior, Theory of planned behavior

## Abstract

**Background:**

Oral health is an important part of public health and crucial to health promotion and enhancing the quality of life. This research examined childhood oral self-care behavior and their related factors using extended Theory of Planned Behavior (TPB).

**Method:**

This cross-sectional study was conducted on 368 sixth-grade elementary school students in Saveh city, Iran, in 2019. The students were selected using a random multi-stage sampling method. The instrument included the socio-demographic questions, TPB constructs, and action and coping plan items. Data were analyzed by SPSS software (Version 21) at alpha level *p* ≤ 0.05.

**Result:**

Overall, 24 (6.6%) students have never used toothbrushes, 222 (62.7%) have never used dental floss, and 298 (82.7%) students have never used mouthwash. The stepwise multiple linear regression analysis results indicated that the TPB with action and coping planning constructs had better predictive power than the original model. In the final model, coping planning (β = .28), intention (β = .24), action planning (β = .23), and perceived behavior control (β = .15) were the most important predictors of oral self-care behavior.

**Conclusion:**

The results indicated that the oral self-care behavior status in Iranian elementary students was not favorable, and the extended model of the TPB with action and coping plan constructs were significant predictors of self-care behavior. Therefore, these findings emphasize the need for expanding educational interventions based on the extended model of the TPB to improve the oral self-care behavior of students.

## Background

Oral health is an important part of public health, in that, it has a major impact on the quality of life, well-being and the overall health of children [1, 2]. A healthy mouth enables a person to eat, talk, communicate better, and socialize [3]. Oral diseases are considered a public health problem worldwide due to their high prevalence and major social impact [4]. Tooth decay is associated with physical, emotional, and economic effects, and not only affects the ability to eat properly and chew, but it can also cause a loss of school hours [4, 5]. Furthermore, physical appearance and diet may worsen and lead to a negative impact on daily life patterns and social relationships [4, 6].

Despite great achievements in the field of oral health worldwide, tooth decay is still a common oral disease in children in many societies, especially in less developed countries [7]. Around the World, 60–90% of schoolchildren and almost 100% of adults have dental cavities [8]. In the United States, 45% of children from ages 5 to 17 years old have decayed teeth [9]. Also, the Decayed, Missing, and Filled Teeth (DMFT) index—which is the sum number of teeth per person that his decayed, missing, or filled and averaged across population--is reported equal to 2.57, 1.30, 2.76, and 1.48 for 12-year-old children in Europe, Africa, USA, and the western Pacific, respectively [9]. According to the report of the Ministry of Health, the amount of DMFT is 1.84 for Iran. In a study by Zandi--Ghashghai et al. (2020), they found that among Iranian 13-year-old students, the mean DMFT was 1.29 (standard deviation [SD] = 1.79), and the rate of untreated tooth decay was 36.7% [10]. According to the Iranian Ministry of Health, most of Iranian students do not pay enough attention to their oral health given that 76.9% brush less than twice a day [11].

Due to the impact of oral health on decreasing oral diseases and improving overall human health, several interventions are available to prevent oral diseases. These interventions include the widespread use of fluoride in various forms, brushing regularly twice a day, flossing at least once a day, changing health habits, regular dental visits, proper diet and reducing sugar intake [12, 13].

Individual oral care, with its emphasis on the personal responsibility for oral health, is a method for preventing cavities and periodontal diseases. In this regard, mechanical methods such as brushing and dental floss are the easiest and most important preventive behaviors to reduce the incidence of dental plaque and prevention of tooth decay. In addition to brushing, South Asian Student Dental Association (SASDA) recommends flossing (or interdental cleaners) once a day to clean teeth [14]. However, adherence to these recommendations among Iranian youth is not very satisfactory [11].

Effective improvement in oral self-care behaviors in society requires an understanding of the underlying factors that influence individuals’ decision-making behaviors [6, 11]. Researchers of socio-psychological science have used various theories to achieve this goal. The Theory of Planned Behavior (TPB) is an effective model in health education that is appropriate for oral health behaviors. This theory provides a good framework for understanding activities that affect oral health [15, 16]. According to this theory, the behavioral intention is the most important determinant of individuals’ behavior. Behavioral intentions are understood as the result of three factors: attitudes, subjective norms, and perceived behavioral control (PBC) [17]. Despite that TPB posits that intention is the most important predictor of behavior, previous studies found that there was a gap between intention and actually performing the behavior [18]. Previous studies supported the entry of planning constructs—or action planning and coping planning--into the TPB [16, 19]. Action planning entails how, when, where the individual will implement the new behavior [15]. For instance, action planning includes not only the individual’s intentions to brush their teeth, but their plan to set an alarm to remind themselves to brush their teeth at a certain time. Coping planning is a barrier-focused strategy [15]. For instance, one may be less likely to brush their teeth in certain situations (e.g., when feeling very tired), and coping planning involves how to perform the intended behavior in these more difficult situations.

Given this prior research, the present study aimed to determine the factors affecting the performance of oral self-care behaviors among Iranian students. Specifically, we examined the associations between oral self-care behaviors and oral care attitudes, subjective norms, and perceived behavior control. In addition, we assessed whether the addition of the constructs of action and coping planning were significantly associated with oral care behaviors.

## Method

### Participants and procedures

In this descriptive study, the statistical population consisted of all sixth-grade students in Saveh, Iran in 2019. The sample size was calculated 304 participants according to the ratio comparison formula and 20% prevalence of oral self-care behavior among this population [20], and considering the 95% confidence level (α = 0.05) with a precision of 0.04. The sample size increased to improve the study precision by 20%, in other words, a total of 368 students entered the study. We selected the students by the random multi-stage sampling from schools with sixth-grade primary grades. At the first stage, we prepared a list of all primary schools of Saveh from two municipal districts. At the next stage, 4 schools (2 girls’ schools and 2 boys’ schools) from each district and thus a total of 8 schools were selected by a simple random method. At the last stage and based on the attendance list, 46 sixth grade students from each school were selected by simple random sampling. The inclusion criterion of the study was that participants were currently enrolled in sixth-grade primary education and written informed consent was obtained from the parents of students. The exclusion criterion included the inability to brush and floss due to physical disability.

In order to complete the questionnaire (see below), we selected the students after coordination and obtaining permission from the Saveh University of Medical Science, school authorities, visiting the primary schools, and coordination with the principals of each school. We explained the research purpose and the method of responding to the questionnaire to the students. We emphasized the confidentiality of the information being gathered and the importance of honest answers. Then, after obtaining informed written consent from the parents of the students we gave the questionnaires to the students. They responded to the questionnaires by the self-report without the presence of teachers in a period of about 50 min.

### Measures

A multi-section self-administered and anonymous questionnaire developed for the current study based on similar studies to collect the data [5, 6, 16].

The first part included five demographic questions and gathered information such as gender, family size, birth rank, and parents’ literacy level.

The second part assessed oral care attitudes with 11 items and questions such as “If I brush my teeth every day, my smile looks more beautiful”. We scored each item on a Likert scale (1 “strongly disagree” to 5 “strongly agree”), and overall scores on oral care attitudes ranged from 11 to 55. Higher scores indicated for favorable attitudes towards oral health behaviors.

The third section assessed Subjective Norms (SN) regarding oral health behaviors and had four questions (e.g., “Most people who are important to me [like my father, mother, friends, teacher, and dentist] think that I should brush my teeth every day”). The scores of this section were also Likert scale (1 “not true at all” to 5 “yes, absolutely true”), with total subjective norms scores ranging from 4 to 20, after reverse coding certain items. Higher scores suggested more perceived social pressure to engage in oral health behaviors.

The fourth section assessed PBC with 5 questions like “I am sure I can floss every night before bed”. Each item was scored on a 5-point Likert scale from “I completely agree” = 5 to “I completely disagree” = 1. Therefore, the range of attainable scores of PBC was a score from 5 to 25. Higher scores indicated that the youth perceived a greater ability or self-efficacy to perform oral health behaviors.

The fifth section assessed intentions to engage in oral health behaviors with 4 questions such as “I am going to use dental floss in the next 2 weeks before going to bed”. Each item was scored on a 5-point Likert scale (1 “very unlikely” to 5 “very likely”). Scores ranged from 4 to 20 with higher scores indicating greater intentions to engage in oral health behaviors.

The sixth section assessed action planning with 4 questions (e.g., “I plan on a time to brush my teeth”) using a five-point Likert scale (1 “completely disagree” to 5 “completely agree”). Scores ranged from 4 to 20 with higher scores indicating greater engagement in action planning for oral health behaviors.

The seventh section assessed coping planning with 8 questions (e.g., “I have a specific program to deal with forgetting the brushing time”) and also used a five-point Likert scale (1 “completely disagree” to 5 “completely agree”). Scores ranged from 8 to 40 with higher scores indicating greater engagement in coping planning for oral health behaviors.

The eighth section measured oral self-care behavior with 4 questions regarding the frequency of brushing, flossing, using mouthwash, and reasons to see a dentist. Scoring in the number of brushing times was as 1 = never to 4 = two or more times daily. For assessing frequency of flossing and mouthwash, respondents indicated: 1 “never” to 3 “once daily.” For assessing reasons for visiting the dentist, respondents indicated: 1 “for pain,” 2 “for checkup,” and 3 for “regular checkup.” A summed total oral self-care behavior score was also calculated and ranged from 4 to 13.

### Validity and reliability

We assessed the overall questionnaire validity using the content validity method. The minimum acceptable amount of content validity rate (CVR) was 0.75 according to the panel of 8 experts. In the content validity index (CVI) estimation stage, 5 items which obtained scores of less than 0.70 were removed [21]. In the next stage, The construct validity of the tool was evaluated by conducting Exploratory Factor Analysis (EFA) and Confirmatory Factor Analysis (CFA). The result of EFA revealed a 7-factor solution containing 40 items (attitude, SN, PBC, intentions, action plan, coping plan and self-care). Average variance extracted of the seven -factor was 0.61. According to CFA results, the seven -factor model showed an acceptable fit (Table 1).

**Table 1:** Goodness-of-fit statistics for model

χ2	P	df	GFI	CFI	TLI	NFI	IFI	RMSEA
286	< 0.001	134	.89	.95	.94	.89	.94	.68

The reliability of the questionnaire was performed through Cronbach’s alpha test with 20 students who were similar to the target population in terms of demographic characteristics but did not participate in the study. The internal consistency of the scale was surveyed using the Cronbach’s alpha coefficients, and for all variables we found acceptable results (attitude =0.88, PBC = 0.84, subjective norms = 0.79, intention = 0.89, action plan = 0.78, coping plan = 0.80 and behavior = 0.75). In Iran, previous studies showed that reliability of the questionnaire has been satisfactory [5, 6, 16].

### Statistical analysis

The Kolmogorov Smirnov test and histogram were employed to determine the normality of data distribution. Inferential statistical methods (independent t-test and one-way analysis of variance) were used for comparisons between groups. Correlation and multiple linear regressions were performed to test the relationships of extended TPB and the oral health-related behaviors. The assumptions of stepwise linear regression evaluated as follows: An analysis of residuals confirmed the assumptions of normality. The results of scatter plot confirmed the assumption of linear relationship between the dependent variable and each of the independent variables. Besides, it should be mentioned that Collinearity was checked and was negative. Data were analyzed using SPSS software Version 22.0 (SPSS Inc., Chicago, IL, USA). *P*-value less than 0.05 at the final stage was considered statistically significant.

## Results

From a total of 368 completed questionnaires, 8 questionnaires were eliminated due to incomplete information. Thus, analyses were performed on 360 questionnaires. The results indicated that the mean age of students was 10.94 (SD = .98). About half of the sample were girls (51.3%), and diploma literacy levels were highest among mothers (*n* = 121) and fathers (*n* = 120), respectively. In terms of birth rank, the highest frequency belonged to the first rank with 146 (40.5%) individuals. Complete demographic information of the sample is reported in Table 2.

**Table 2:** Basic characteristics of the participants (*n* = 360)

Characteristics	n	%
Sex		
Male	175	48.7
Female	185	51.3
Father’s education		
Illiterate/ Elementary	67	18.6
Middle school	69	19.1
High school and diploma	120	33.3
University	104	28.9
Mother’s education		
Illiterate/ Elementary	89	24.7
Middle school	77	21.4
High school and diploma	121	33.6
University	73	20.3
Birth rank		
1	146	40.6
2	89	24.7
3	81	22.5
≥ 4	44	12.2
Socio-economic status		
good	208	57.8
medium	138	38.3
weak	14	3.8

As Table 3 illustrates, 24 (6.6%) students reported having never brushed their teeth, and 76 (21%) reported brushing their teeth twice or more a day. Regarding the use of dental floss and mouthwash, the results indicated that 222 (62.7%) and 298 (82.7%) individuals never used dental floss and mouthwash, respectively. Eighty-five (23.6%) reported that they visited the dentists for a toothache.

**Table 3:** Frequency distribution of oral health behaviors among studied students

behavior		
brushing	Number	%
never	24	6.7
sometimes	125	34.7
once daily	135	37.6
two or more on a daily	76	21
Dental floss		
never	226	62.8
sometimes	28	7.8
once daily	106	29.4
Preventive mouthwash		
never	298	82.7
sometimes	40	11.1
once daily	22	6.2
visits to dentist		
In pain	85	23.6
Check up	189	52.5
Regular check up	86	23.9

The ANOVA analyses revealed that mean scores of all constructs of TPB and coping and action planning, except for subjective norms, were significantly higher in students, who brushed twice or more a day compared to students who brushed once a day, sometimes and never. Regarding flossing and using mouthwash, the mean scores of all constructs of the TPB model were significantly higher in students who flossed daily and used mouthwash than students who had reported sometimes or never engaging in those behaviors (*p* < 0.05). There were no significant differences in TPB construct scores when comparing different reasons for visiting the dentist (*p* > 0.05). Results from ANOVA analyses can be found in Tables 4 and 5.

**Table 4:** Distribution of mean and standard deviation scores of model constructs based on brushing

behavior	never	sometimes	once daily	two or more on a daily	df	f	η2
brushing	Mean ± SD	Mean ± SD	Mean ± SD	Mean ± SD			
Attitude	16.9 ± 4.1	18.9 ± 6.0	20.1 ± 6.2	23.1 ± 6.8	26	1.94*	.13
SN	4.3 ± 1.7	4.6 ± 2.1	5.5 ± 2.5	5.7 ± 2.2	10	.55	.01
PBC	12.7 ± 6.1	12.9 ± 3.7	14.6 ± 3.3	16.2 ± 4.6	21	4.32*	.21
Action plan	7.5 ± 3.8	8.0 ± 3.7	10.7 ± 3.5	11.9 ± 3.4	18	4.43*	.19
Coping plan	13.1 ± 6.1	13.4 ± 6.7	16.0 ± 6.8	18.2 ± 7.2	29	2.85*	.20
intention	6.6 ± 3.0	7.6 ± 4.8	12 ± 4.6	13.9 ± 4.1	16	1.73*	.07

*.p < 0.05

**Table 5:** Distribution of mean and standard deviation scores of model constructs based on oral health behaviors

behavior	never	sometimes	once daily	df	f	η2
Dental floss	Mean ± SD	Mean ± SD	Mean ± SD			
Attitude	17.6 ± 5.3	18.8 ± 6.3	20.5 ± 7.1	26	1.82*	.12
SN	4.8 ± 2.0	5.1 ± 2.4	5.8 ± 3.2	10	1.81*	.04
PBC	12.2 ± 4.1	14.1 ± 4.1	16.5 ± 4.2	21	7.53*	.31
Action plan	8.1 ± 4.2	9.2 ± 4.5	9.5 ± 5.2	18	41.37*	.68
Coping plan	13.6 ± 6.2	14.1 ± 6.6	16.1 ± 7.2	29	5.48*	.32
intention	6.7 ± 4.5	7.8 ± 4.8	11.7 ± 4.2	16	3.92*	.15
Preventive mouthwash						
Attitude	19.0 ± 6.3	19.9 ± 6.2	24.5 ± 6.0	26	1.84*	.12
SN	4.6 ± 2.1	5.1 ± 2.5	5.8 ± 2.1	10	3.16*	.08
PBC	13.1 ± 3.7	13.6 ± 4.3	16.0 ± 4.9	21	5.50*	.25
Action plan	7.5 ± 3.9	8.8 ± 3.5	9.1 ± 4.8	18	3.00*	.13
Coping plan	13.0 ± 6.2	14.3 ± 5.8	15.4 ± 6.9	29	2.86*	.20
intention	6.9 ± 4.6	7.9 ± 5.1	9.4 ± 4.4	16	3.19*	.13
visits to dentist						
Attitude	19.0 ± 6.2	19.2 ± 4.1	19.6 ± 5.7	26	1.33	.09
SN	4.7 ± 2.2	5.3 ± 2.5	5.4 ± 2.5	10	1.14	.03
PBC	13.3 ± 4.2	13.5 ± 4.2	15.0 ± 4.1	21	1.28	.07
Action plan	8.3 ± 4.5	8.6 ± 3.6	9.3 ± 4.4	18	1.13	.05
Coping plan	13.0 ± 4.9	14.1 ± 6.8	15.6 ± 6.6	29	1.00	.08
intention	7.1 ± 4.9	7.5 ± 4.1	8.8 ± 5.6	16	1.31	.09

*.p < 0.05

The Pearson correlation coefficient test showed that all constructs of the TPB, including action planning and coping planning variables, had a positive and significant correlation with overall oral self-care behavior scores. Intentions to engage in oral care behaviors had the strongest correlation (*r =* .52) to oral self-care behaviors, and the attitude towards oral care behaviors scores had weakest correlation with behavior respectively (*r =* .31; *see* Table 6).

**Table 6:** TPB Variables, action and coping plan with oral health behavior correlation matrix

variable	Attitude	Subjective norm	PBC	Action plan	Coping plan	Intention	Behavior
**Attitude**	**1**						
**Subjective norm**	0.14**	1					
**PBC**	0.35**	0.09	1				
**Action plan**	0.32**	0.13*	0.55**	1			
**Coping plan**	0.41**	0.39**	0.44**	0.70**	1		
**Intention**	0.34**	0.46**	0.53**	0.47**	0.49**	1	
**Behavior**	0.31**	0.34**	0.49**	0.44**	0.47**	0.52**	1

* P < 0.05 **P **< 0.01**

The stepwise multiple linear regression analysis results indicated that the PBC and intention constructs were significant predictors of oral self-care behavior; and TPB constructs accounted for 25% of variance in oral self-care behavior scores. In the second step, AP and CP were added to the TPB model (Adjusted *R*-Square = .29). The results indicated that when action planning and coping planning constructs were added in step 2, the new model had significantly better predictive power than the original model. In the final model, coping planning (β = .28), intention (β = .24), action planning (β = .23), and PBC (β = .15) were the most associated with oral health behaviors (Table 7).

**Table 7:** Results obtained from Multiple Linear Regression: TPB constructs, and Action plan and Coping plan predicting oral self-care behavior scores

	Unstandardized Coefficients		Standardized β	t	95%confidence interval for B		*P* Value
	B	SE			Lower Bound	Upper Bound	
Step 1							
**Attitude**	0.05	0.03	0.10	1.60	−0.01	0.12	0.10
**Subjective norm**	0.32	0.22	0.08	1.47	−0.11	0. 76	0.14
**PBC**	0.71	0.26	0.16	2.67	0.18	1.23	0.008
Intention	0.34	0.06	0.29	5.21	0.21	0.47	0.001
**Model Adjusted R Square = 0.25**							
Step 2							
**Attitude**	0.007	0.03	0.01	0.20	−0.06	0.07	0.84
**Subjective norm**	0.02	0.31	0.005	0.07	−0.58	0.63	0.94
**PBC**	0.58	0.28	0.15	2.05	0.02	1.15	0.04
Intention	0.28	0.06	0.24	4.38	0.15	0.41	0.001
**Action plan**	0.22	0.07	0.23	2.94	0.07	0.37	0.003
**Coping plan**	0.18	0.05	0.28	3.21	0.07	0.29	0.001
**Model Adjusted R Square = 0.29**							

## Discussion

The focus of the present study was on assessing the predictive power of the TPB model, including the constructs of action planning and coping planning, on oral self-care behavior. Our findings suggest that the two action planning and coping planning increased the predictive power of the model. In line with our findings, previous studies supported adding action and coping planning to the TPB as useful constructs in predicting oral self-care behavior [16, 22, 23]. For instance, both Schwarzer et al. [24] and Zhou et al. [25] concluded that the two constructs were important motivational variables for the formation of behavior. These findings emphasized that successful implementation of oral self-care behavior required creating a plan for the time, place, and way of completing the behavior, as well as establishing a self-regulatory strategy to manage competing goals and barriers to intended behavior. Past studies suggest that action planning plays an important role in initiating and normalizing a behavior by including it into everyday tasks; and that coping planning seems necessary to maintain a behavior because by anticipating obstacles [16, 19].

In our study, intention and PBC were significant predictors of oral self-care behavior. In the TPB (theory of planned behavior), the intention is the immediate determinant of behavior. PBC reflects the students’ perception of how difficult it may be to engage in behaviors such as brushing and flossing. Previous studies in the field of oral health have proven that PBC is one of the most important factors influencing behavioral intention and behavior [15, 26]. Consistent with the present study, Naseri-Salahshour et al. found that an oral health intervention that target perceived behavioral control was efficacious in increasing tooth brushing among a sample of elementary school students in Iran [5].

Our findings indicated that a significant percentage of students (41.4%) brushed their teeth less than once a day. Consistent with this result, Ashoori et al. [7] and Soltani et al. [27] found that 35 and 34.5%, respectively, of Iranian youth reported brushing their teeth less than once a day. Studies conducted on adolescents in other regions of the world indicate better rates of toothbrush use compared to Iranian adolescents. For instance, the rate of those reporting brushing their teeth less than once a day was only 10.3% in study of Romanian adolescents [28], 1.4% in Sweden adolescents [25], 14.2% in Laos [29], and 14% in a study of young children in the United States [30]. Our finding regarding low rates of oral self-care should be seriously taken into consideration by health policymakers given that brushing is one of the most important ways to prevent oral diseases.

In our study, about a third of students reported daily use of dental floss. Consistent with our findings, daily flossing by Iranian students was reported by Pourhaji et al., [31] and Soltani et al., [27] to be 44.8 and 31%, respectively. Compared to other countries, the rate was 19.6% in a study by Farsi in Saudi Arabia [32], 13.1% in Japan [33], 7.3% in Nigeria [34], and 10.6% in Egypt [35]. Another recent study in Saudi Arabia reported that almost 0% reported use of dental floss [36]. It seems that the use of dental floss is low in Iran and in many Asian and African countries. Educational and cultural measures may be necessary to increase oral health in these regions.

Our results showed that 17.2% of students reported using mouthwash. Bayat et al. [37] and Alami et al. [38] reported similar rates in Iranian samples. As compared to other regions in the world, the rate of mouthwash use was 48% in a study in India [39], 58% in Pakistan [40], and 35.6% in Saudi Arabia [32]. Due to the low use of mouthwash and because fluoride has not been added to drinking water in Iran, the frequent use of sodium fluoride mouthwash to help reduce tooth decay.

Furthermore, in our study, 23% of students went to the dentist when they were in pain and 23.8% went to regular checkups. There was no significant difference in scores on the TPB constructs in terms of reasons to see a dentist. This finding may be explained by the possibility that the dentists did not provide adequate oral health training for students. Contrary to our findings, several studies [32] reported that toothache as the main reported reason for going to the dentist. A likely reason for this difference may be attributed to the implementation of the dental health system development plan in recent years in Iran which included mandatory dental visits for students in the first and sixth grades of primary school.

## Conclusion

The results indicated that the oral self-care behavior status in Iranian elementary students was not favorable, and the extended model of the TPB with action and coping planning constructs were significant predictors of oral self-care behavior. Therefore, these findings emphasize the need for expanding educational interventions based on the extended model of the TPB to improve the oral self-care behavior of students.

## Data Availability

All necessary data are presented within the manuscript. All other material send data are available upon request.

## Abbreviations

TPB: Theory of Planned Behavior
PBC: perceived behavior control
CP: coping planning
AP: action planning
DMFT: Decayed, missing, and filled teeth
CVR: content validity rate
CVI: content validity index
